# Minor Surgery and Bandaging

**Published:** 1894-02

**Authors:** 


Minor Surgery and Bandaging. By Henry R. Wharton, M. D., Demonstrator of Surgery in the University of Pennsylvania. In one 12mo volume of 529 pages, with 416 engravings, many being photographic. Cloth, $3.00. Philadelphia, Lea Brothers & Co., 1893.

An excellent book for students and young practitioners.

The author has presented clearly and concisely a description of the various bandages and surgical dressings employed; the preparation and uses of the most commonly employed antiseptic dressings together with description of the method for reduction of the various fractures and dislocations.

The cuts are numerous and well made, being in most instances taken from photographs. Short articles on Tracheotomy, Intubation of Larynx, Ligation of Arteries and Amputations, constitute a valuable addition to the work, and we commend it heartily to the medical student.

